# Deciphering the role of insertion sequences in the evolution of bacterial epidemic pathogens with *panISa* software

**DOI:** 10.1099/mgen.0.000356

**Published:** 2020-03-26

**Authors:** Charlotte Couchoud, Xavier Bertrand, Benoit Valot, Didier Hocquet

**Affiliations:** ^1^​ Laboratoire d’Hygiène Hospitalière, Centre Hospitalier Régional Universitaire, Besançon, France; ^2^​ UMR CNRS 6249 Chrono-environnement, Université de Bourgogne Franche-Comté, Besançon, France; ^3^​ Bioinformatique et big data au service de la santé, UFR Santé, Université de Bourgogne Franche-Comté, Besançon, France

**Keywords:** whole-genome sequencing, outbreak, insertion sequence, bacterial evolution

## Abstract

Next-generation sequencing (NGS) is now widely used in microbiology to explore genome evolution and the structure of pathogen outbreaks. Bioinformatics pipelines readily detect single-nucleotide polymorphisms or short indels. However, bacterial genomes also evolve through the action of small transposable elements called insertion sequences (ISs), which are difficult to detect due to their short length and multiple repetitions throughout the genome. We designed *panISa* software for the *ab initio* detection of IS insertions in the genomes of prokaryotes. *PanISa* has been released as open source software (GPL3) available from https://github.com/bvalot/panISa. In this study, we assessed the utility of this software for evolutionary studies, by reanalysing five published datasets for outbreaks of human major pathogens in which ISs had not been specifically investigated. We reanalysed the raw data from each study, by aligning the reads against reference genomes and running *panISa* on the alignments. Each hit was automatically curated and IS-related events were validated on the basis of nucleotide sequence similarity, by comparison with the ISFinder database. In *Acinetobacter baumannii *, the *panISa* pipeline identified IS*Aba1* or IS*Aba125* upstream from the *ampC* gene, which encodes a cephalosporinase in all third-generation cephalosporin-resistant isolates. In the genomes of *Vibrio cholerae* isolates, we found that early Haitian isolates had the same ISs as Nepalese isolates, confirming the inferred history of the contamination of this island. In *Enterococcus faecalis *, *panISa* identified regions of high plasticity, including a pathogenicity island enriched in IS-related events. The overall distribution of ISs deduced with *panISa* was consistent with SNP-based phylogenic trees, for all species considered. The role of ISs in pathogen evolution has probably been underestimated due to difficulties detecting these transposable elements. We show here that *panISa* is a useful addition to the bioinformatics toolbox for analyses of the evolution of bacterial genomes. *PanISa* will facilitate explorations of the functional impact of ISs and improve our understanding of prokaryote evolution.

## Data Summary

We confirm that all supporting data, code and protocols have been provided within the article or through Supplementary Material.

**Impact Statement**

Insertion sequences (ISs) are small transposable elements playing a key role in bacterial genome organization and evolution. They are difficult to detect in sequencing data. We therefore designed *panISa* software for the *ab initio* detection of IS insertion in prokaryotic genomes. Here, to evaluate the potential of this new tool for use in evolutionary studies, we selected five published studies describing genome evolution in five major human epidemic pathogens. None of these studies had used bioinformatics pipelines to retrieve ISs. The ISs retrieved by *panISa* had a genomic distribution consistent with SNP-based phylogenetic analysis. Our pipeline rapidly detected IS-related mechanisms of resistance to antibiotics and identified genomic regions of high plasticity with a high concentration of IS-related events. The proportion of genomes displaying IS insertions varied considerably between bacterial species, but was at least 86 % for *Acinetobacter baumannii *, *Vibrio cholerae *, and *Enterococcus faecalis. PanISa* is a useful addition to the bioinformatics toolbox for analyses of prokaryote evolution. It will help us to determine the role of ISs in pathogen evolution, which has probably been underestimated.

## Introduction

Whole-genome sequencing (WGS) is becoming the gold-standard technique for investigating the evolution of bacterial pathogen genomes during their spread. Application of the appropriate pipelines to sequencing data results in the detection of single-nucleotide polymorphisms (SNPs) or small insertion/deletion (indels) after the alignment of reads with a reference genome sequence. However, bacterial genomes also evolve through the insertion of insertion sequences (ISs), which are widespread and occur in all domains of life [1]. ISs are mobile autonomous elements formed by (*i*) one or two transposase-encoding genes, (*ii*) two terminal inverted repeats (IRs), and (*iii*) two direct repeated sequences (DRs) [2]. ISs are sorted into families using the amino acid similarity of their transposase [3]. In 2019, the ISFinder database reported more than 4000 ISs belonging to 29 families [2, 3]. Genes are inactivated by the insertion of an IS into their coding sequences. ISs can also modulate the expression of a gene if they disrupt its promoter or create an alternative promoter [4]. Most known examples of IS transposition are linked to antibiotic resistance, because the resulting phenotypes are easy to detect. For example, the insertion of IS*1* or IS*10* upstream from the efflux pump gene *acrEF* increases the resistance of *Salmonella enterica* to fluoroquinolones [5]. Similarly, the insertion of IS*Aba1* or IS*Aba125* upstream from *ampC* increases resistance to third-generation cephalosporin in *Acinetobacter baumannii* [6, 7]. This ability of IS insertion to affect bacterial resistance to antibiotics and virulence can help bacterial pathogens to adapt to new niches [4]. IS dynamics are rarely investigated, but an understanding of these dynamics during outbreaks of pathogenic prokaryotes could be highly informative in evolutionary studies.

The detection of ISs is challenging, because read lengths are usually shorter (<300 bp) than ISs and the same IS may be repeated in the genome. We have developed the *panISa* program to detect new and unknown insertions *ab initio* (i.e.

with a database-free approach) in bacterial genomes, based on the detection of structural variants in short-read data. *PanISa* requires only short reads and a reference genome as input. The presence of ISs and their repeated nature renders the IS localization from the assembly very challenging, which is why we used a software that localizes ISs from the raw reads. The program has been validated on simulated data and compared with existing tools, but the benefits of IS detection for epidemiological studies remain to be evaluated [8].

We therefore assessed the dynamics of IS insertion during the spread of bacterial pathogens, by reanalysing the WGS data from five published studies describing genome evolution for major epidemic bacterial pathogens.

## Methods

### Selection of the datasets

We reanalysed five published datasets from studies aiming to decipher the evolution of a speciﬁc clone of a bacterial pathogen during its spread (Table 1). All the species concerned were major human pathogens for which ISs have already been described.

**Table 1.** Collections of genomes of bacterial pathogens reanalysed with panISa software

Reference	Reference genome (NCBI accession numbers)	Isolates *(n)*	
		Total no.	Isolates with available data
Eppinger *et al*. [9]	* Vibrio cholerae* O1 biovar El tor str. N16961 (AE003852.1; AE003583.1)	116	110
Martinez-Urtaza *et al*. [10]	* Vibrio parahaemolyticus* RIMD 2210633 (BA000031.2; BA000032.2)	48	16
Raven *et al*. [11]	* Enterococcus faecalis* V583 (AE016830.1)	168	168
Wilson *et al*. [12]	*Salmonella Tennessee* str. TXSC_TXSC08-19 (CP007505.1)	69	68
Holt *et al*. [13]	* Acinetobacter baumannii* strain A1 (CP010781.1)	44	35

The first dataset was from a study describing the global epidemiology of the seventh cholera pandemic that aimed to identify the geographic origin of the contamination of Haiti [9]. We also selected a dataset for isolates of the transcontinental epidemic strain of *Vibrio parahaemolyticus* ST36 [10]. The authors reconstructed the evolution of this clone over the last 25 years, by genome-wide analysis. We selected a dataset for genomes from a global collection of isolates of *Enterococcus faecalis* retrieved between 1958 and 2012 from bloodstream infections in Ireland, the UK and the USA [11]. Another collection of genomes of *Salmonella enterica* subspecies *enterica* serotype Tennessee (*S*. Tennessee) isolates from an outbreak involving transmission in peanut butter was also selected [12]. In this study, SNP-based analysis revealed that the contamination was of environmental origin. Finally, we selected a dataset of genomes of isolates of *Acinetobacter baumannii* global clone 1 (GC1) collected between 1960 and 2011, the analysis of which provided insight into the evolution of these genomes and the phylodynamics of GC1 [13].

### Downloading and pretreatment of short-read data

For the five datasets, we first determined the number of isolates for which short-read data were available in the sequence read archive (SRA) database (1. Eppinger *et al.* [9], 2. Martinez-Urtaza *et al.* [10], 3. Raven *et al.* [11], 4. Wilson *et al.* [12], 5. Holt *et al.* [13]) ((Table 1; Data S1, available in the online version of this article). We used prefetch and fastq-dump command-line tools from NCBI toolkits to extract and convert the data into an optimized input format for *panISa* [14]. Reads were subsampled to a final coverage of 60× and mapped against the same reference genome as in the original studies with the Burrows–Wheeler aligner [15].

### PanISa search

*PanISa* identifies IS insertions through comparison with the pysam library. Briefly, each read that maps partially on the reference genome was detected by *panISa* as a clipped read. When clipped reads were in opposite directions on two close positions of the genome, the program identified a potential IS insertion (also called a hit). The flanking parts of the clipped reads defined the boundaries of the insertion, with start clipped reads defining the IRR and end clipped reads defining the IRL (see Treepong *et al.* [8] for a detailed description of the functioning of the software). *PanISa* has been released as open-source software (GPL3) available from https://github.com/bvalot/panISa. Each alignment ﬁle (.bam) was used as input for *panISa* with the minimum clipped reads option set to 10 (default settings for all other options). As *panISa* detects all insertion events, manual curation of the list of potential ISs is required. We checked for sequence similarity (identity >90 % over>80 % of the length of the sequence) between the boundaries of the IS (IRR and IRL), with the ISFinder database, to conﬁrm IS insertions [16]. To clarify the terms used thereafter, *panISa* produces a ‘hit’ for each detection of whichever insertion, while an ‘IS-related event’ describes the insertion of an IS. In other words, if one specific IS inserted at two different positions, we described it as one IS and two IS-related events.

### Estimation of the biological impact of IS insertion

We downloaded the annotations (i.e. GFF general feature format) of each reference genome to identify the function of the proteins encoded by the genes disrupted by ISs. In addition, for ISs inserted into intergenic regions, we identified the function of the proteins encoded by genes with translation start sites <100 bp away from the insertion site of an IS.

## Results and discussion

In this study, we used *panISa* to reanalyse published WGS datasets. Unsurprisingly, none of these published studies could identify new ISs or new IS insertion sites. However, Holt *et al.* [13] reported the detection of IS*Aba1* and IS*Aba125* upstream from the cephalosporinase-encoding *ampC* gene by a PCR approach for the identification of genetic events leading to antibiotic resistance [13]. ISs can contribute to antibiotic resistance and virulence, depending on the nature of the genes that are disrupted or imported. They can also, more widely, contribute to bacterial genome rearrangements. The identification of IS-related events with an accurate tool would therefore improve our understanding of genome evolution in epidemic pathogens.

### IS detection in the genomes of epidemic bacterial pathogens

*PanISa* retrieved 692 to 15 878 hits from the five datasets, the smallest number of hits being obtained for the study of *V. parahaemolyticus* genomes and the largest number for the study of *E. faecalis* (Table 2). Only hits matching sequences in the ISFinder database were considered to be IS-related events. Thus, 207 of the 2913 hits were identified as IS-related events in the *V. cholerae* dataset [9]. *PanISa* identiﬁed 1348 IS-related events among the 15 878 hits for the *E. faecalis* dataset and 345 IS-related events among the 1371 hits for the *A. baumannii* dataset [11, 13]. None of the 692 hits for the *Vibrio parahaemolyticus* dataset was associated with an IS [10]. We identified four IS-related events among the 727 hits for *S.* Tennessee, all involving the same IS in a single isolate [12]. The small numbers of IS-related events detected in these last two datasets may be due to either the short length of the reads (<100 bp) creating artefactual repeat sequences or the low coverage (<40×) for several genomes [8]. Furthermore, as Wilson *et al.* [12] studied genomes of *S.* Tennessee isolates collected between 2006 and 2008, the study period may have been too short for IS insertion to happen [12] (Data S2). In addition, the short length of the reads or the presence of repeated regions may account for the high level of background noise.

**Table 2.** Result of the reanalysis of five genome collections with *panISa*.

Reference	Species	*panISa* hits (*n*)	IS-related events (*n*)	ISs (*n*)	Proportion (%) of isolates with ≥1 IS
Eppinger *et al.* [9]	* Vibrio cholerae *	2913	207	5	91
Martinez-Urtaza *et al.* [10]	* Vibrio parahaemolyticus *	692	0	0	0
Raven *et al.* [11]	* Enterococcus faecalis *	15 878	1348	29	100
Wilson *et al.* [12]	* Salmonella* Tennessee	727	4	1	1.4
Holt *et al.* [13]	* Acinetobacter baumannii *	1371	345	19	86

The third column shows the number of insertion events identified by *panISa* from WGS datasets of bacterial pathogens, the fourth column gives the number of hits matching sequences in the ISFinder database, considered to correspond to IS-related events, the fifth column gives the number of different ISs found among the IS-related events, and the last column gives the proportion of isolates concerned.

### Phylogeny of the *Vibrio cholerae* outbreak

**Fig. 1.**
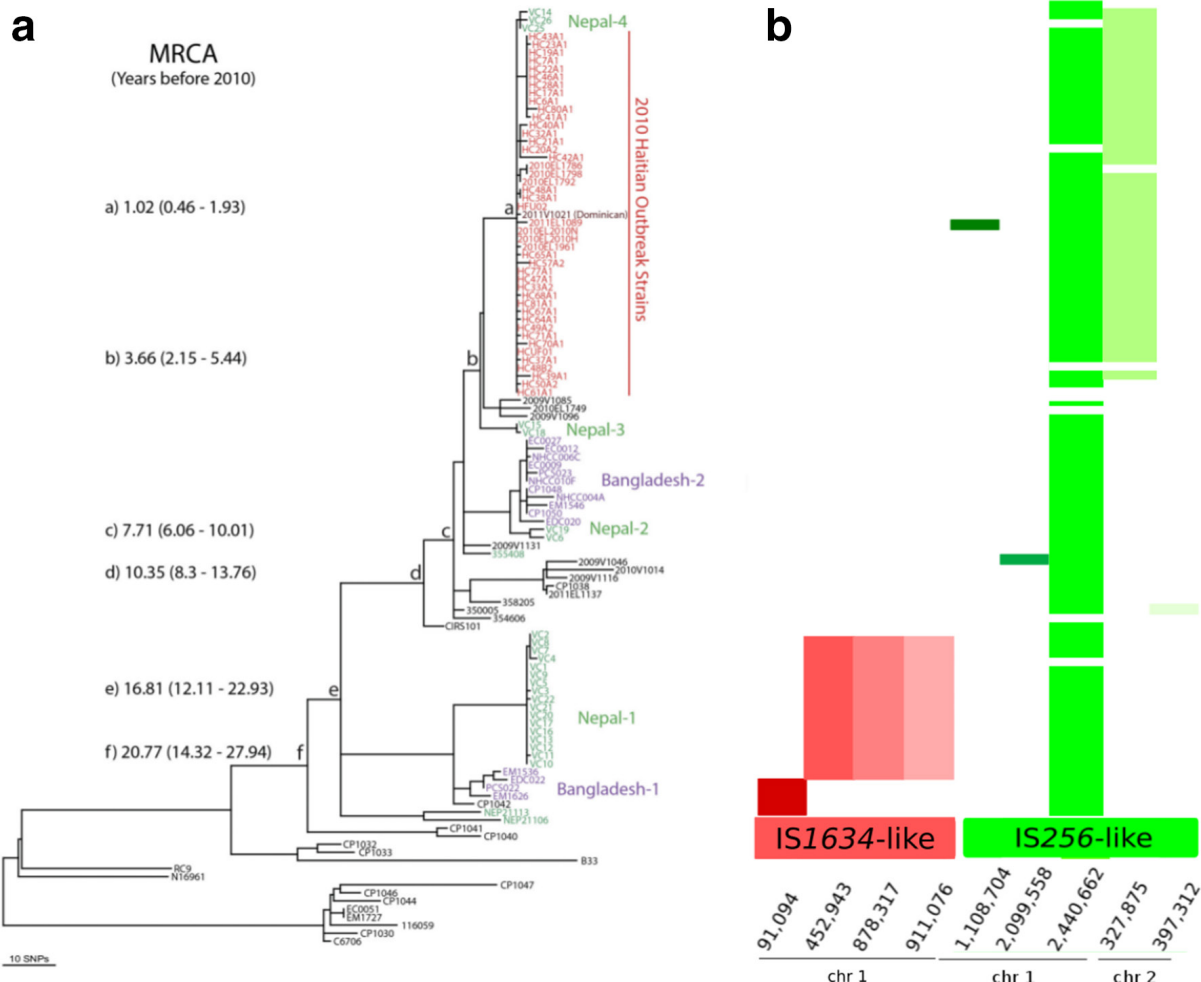
Comparison of (a) phylogenetic analysis from Eppinger *et al.* [9] and (b) IS-related events identified by *panISa*. Red boxes represent insertions of IS*1634*-like elements and green boxes represent insertions of IS*256*-like elements. Each column represents an insertion site on a specific chromosome. The two colours refer to the two different ISs detected in the dataset, and the different shades of colours represent the different positions of insertion of each IS [at the chromosomal positions indicated at the bottom of (b)].

Haiti had been free from *V. cholerae* for 100 years before being hit, in 2010, by an outbreak that lasted 2 years. Phylogenetic analysis revealed that Haiti had been contaminated by a Nepalese strain brought to the island by United Nations peacekeepers [17]. We reanalysed datasets of genome sequences for *V. cholerae* isolates from Haiti, Nepal and Bangladesh [9]. Phylogenetic analysis of these sequences performed by Eppinger *et al.* [9] revealed these isolates to be highly homogeneous. *PanISa* identified 207 insertions of five different ISs. The IS*256*-like element, inserted at position 2 440 662, was common to all isolates of the dataset. The IS*256*-like element in position 327 875 was specific to the genomes of isolates from cluster ‘a’, which grouped together isolates obtained in Haiti in 2010 and isolates from Nepal (Fig. 1a). It is unlikely that independent insertions of the same IS occurred at the same genomic position, so the insertions of the IS*256*-like element probably occurred in a common ancestor of the Haitian isolates and the Nepalese isolates of the ‘Nepal-4’ subcluster (Fig. 1a). This IS insertion pattern is consistent with the close relationship showed by the phylogeny between the Haitian and Nepalese isolates.

Similarly, the IS*1634*-like element inserted at position 91 094 was speciﬁc to isolates clustering in the ‘Bangladesh-1’ group, and the three IS*1634*-like elements inserted at positions 452 943–878 317 and 911 076 were speciﬁc to isolates clustering in the ‘Nepal-1’ group, as defined by Eppinger *et al.* [9]. These insertions undoubtedly occurred in a common ancestor and were then transmitted to all isolates derived from that ancestor (Fig. 1b).

The localization, by *panISa,* of the IS insertions in the genomes made it possible to identify the genes affected and to predict the biological consequences of the gene disruption or the modification of gene expression. Unfortunately, the disrupted genes encoded proteins did not seem to be involved in known virulence or resistance (Data S3), making it difficult to implicate any of these changes in the development of antibiotic resistance or virulence during the spread of *V. cholerae *. However, the identification of IS insertions with *panISa* provided information consolidating the phylogenetic trees built from SNP data.

### IS insertions in the antibiotic-resistant *Acinetobacter baumannii* global clone 1

**Fig. 2.**
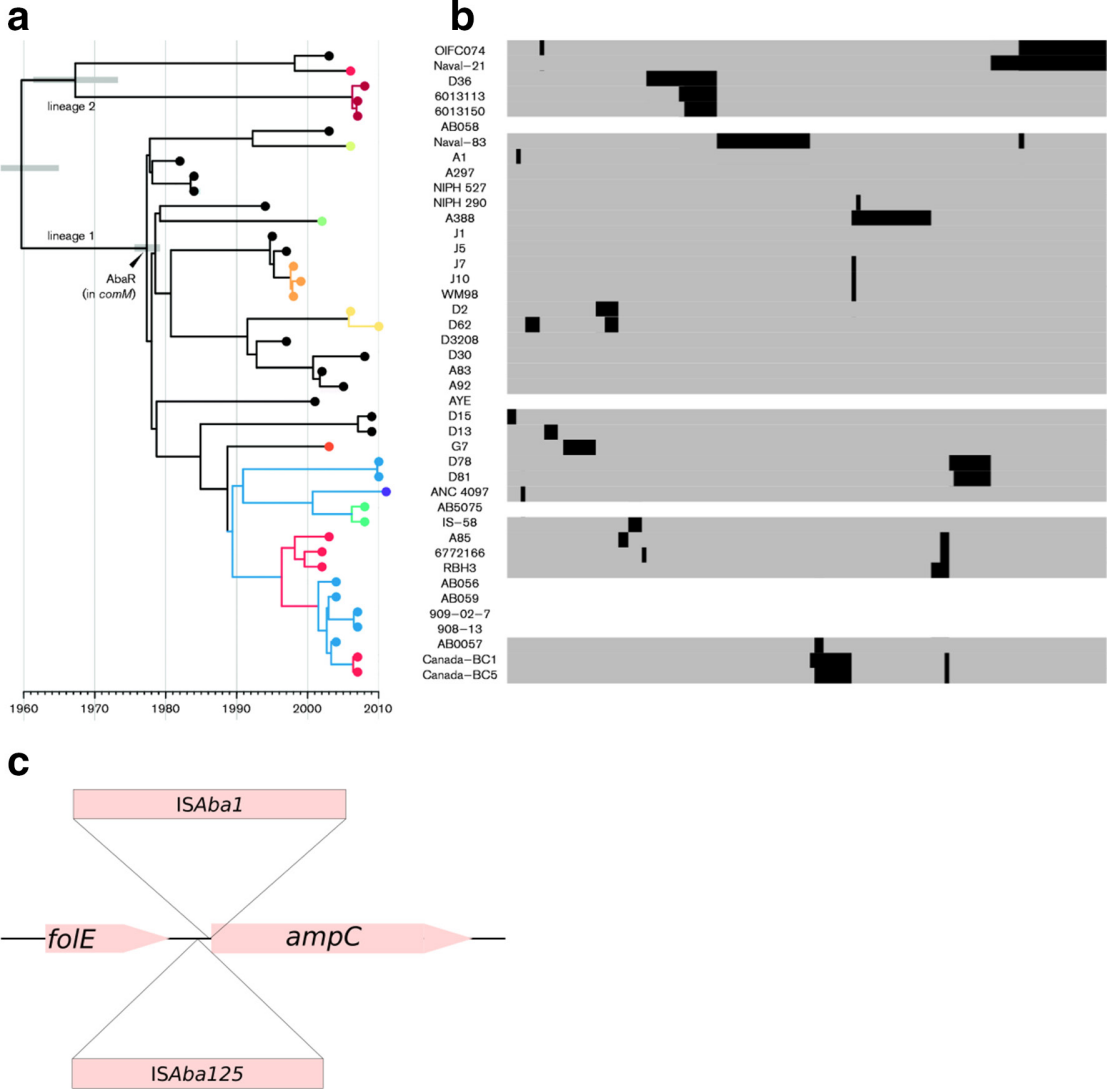
Phylogenic analysis of *A. baumannii* GC1. (a) Temporal and phylogenetic analysis performed by Holt *et al.* [13]. The dot colours represent the capsule type of each isolate. (b) Hierarchical clustering based on the presence/absence of 345 IS-related events implicating 19 ISs in 35 isolates of *A. baumannii* with available SRA data. Each of the 345 columns represents one IS-related event, with grey indicating no event, black indicating an event and white if the sequence data could not be obtained. (c) Representation of the insertion of IS*Aba1* and IS*Aba125* upstream from ampC.

We reanalysed the *A. baumannii* genome dataset with *panISa* and retrieved 345 IS-related events in 35 genomes, with 86 % of the genomes displaying at least one IS-related event [13] (Table 2; Data S4). The wide temporal coverage of the collection (~50 years) probably accounts for the large number of IS events retrieved (Fig. 2a). Likewise, the isolates were selected to represent the maximum diversity among the global clone 1, the high variability of sources and locations presumably accounted for the higher number of IS-related events detected. We performed hierarchical clustering based on the presence/absence of the 345 IS-related events, which revealed similarities between the isolates (Fig. 2b). Similar IS-related event profiles were observed in some clusters of two or three isolates, such as Canada-BC1 and Canada-BC5, and isolates D78 and D81. The global results for IS-based clustering were consistent with those based on SNP data reported by Holt *et al.* [13] (Fig. 2a).

The original study aimed to identify the determinants of resistance to third-generation cephalosporins. The insertion of an IS upstream from the cephalosporinase-encoding *ampC* gene can lead to the overproduction of AmpC, increasing resistance to third-generation cephalosporins [6, 7]. The authors of the original study used PCR and sequencing to search for ISs upstream from *ampC*, but *panISa* rapidly and correctly identiﬁed all the IS*Aba1* and IS*Aba125* insertions upstream from *ampC* in all 13 AmpC*-*overproducing isolates (Fig. 2c) [13]. Thirteen of the 19 different ISs retrieved by *panISa* had already been described in this pathogen [16]. The proportion of isolates displaying at least one IS-related event was consistent with previous reports [18]. The most active IS in the global clone was IS*Aba1,* with 7 % of its insertions disrupting the genome upstream from *ampC* [18]. We found that the most common position for insertions in these genomes was upstream from an IS*256* (after nucleotide 287 817) element already present in the reference genome.

Thus, *panISa* is a useful tool for identifying IS insertions with a well-described biological impact, such as antibiotic resistance. *PanISa* can not only retrieve IS insertions in well-known location (i.e. upstream from *ampC*) but also everywhere in an Illumina-sequenced genome. *PanISa* can accelerate the laborious task of targeted searches for ISs in genomes. Our genomic data also indicate that this pipeline can detect unexpected insertion sites, thereby improving our understanding of the genomic events leading to particular phenotypes.

### Distribution of IS insertions in the genome of *Enterococcus faecalis*

**Fig. 3.**
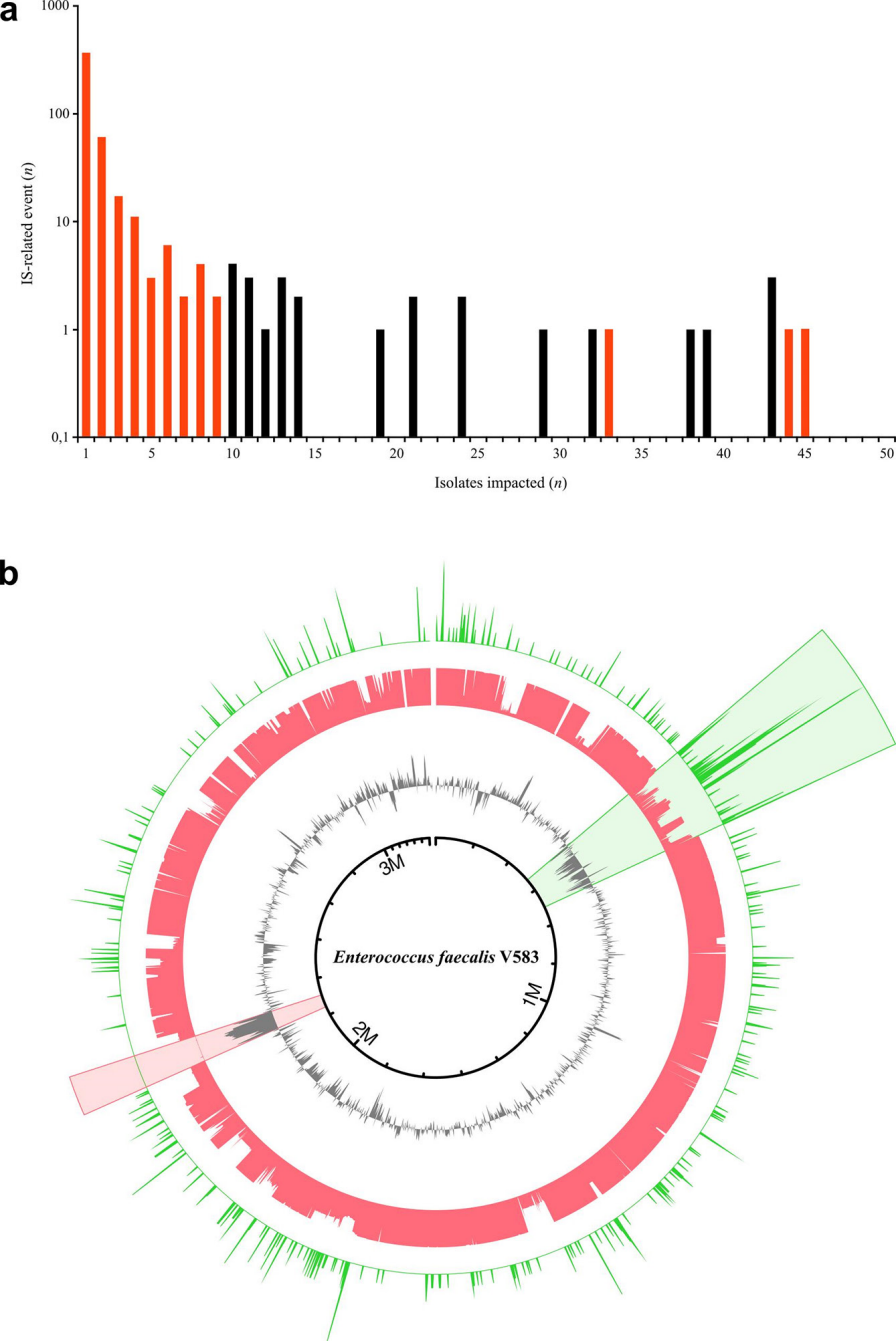
Distribution of the IS-related events in the genomes of a collection of 168 clinical isolates of *E. faecalis* [11]. (a) Distribution of the number of IS-related events as a function of the number of isolates affected. The *y*-axis is drawn to a logarithmic scale. Red bars represent IS-related events that occurred >100 bp from a coding sequence or occurred in less than ten isolates. Black bars represent IS-related events that occurred within 100 bp of a coding sequence and are reported in Table 3. (b) The outer green circle represents the number of IS-related events, the red central circle indicates the number of genomes from the collection of 168 isolates of *E. faecalis* containing the region, and the inner grey circle represents GC-content relative to the mean value. The 150 kb pathogenicity island and the ICE (ICEEEfaV583-1) of *E. faecalis* V583 are indicated by the green and red sectors, respectively.

We reanalysed the data for an international collection of isolates of *E. faecalis* from bloodstream infections collected over the last 50 years [11]. *PanISa* retrieved 1348 IS-related events at 472 different genomic sites. All of the 168 genomes for which SRA data were available presented at least one IS insertion (Table 2). More than a third of the IS insertions involved IS*Enfa4*, an *E. faecalis* IS from the IS*256* family. The large number of ISs identiﬁed presumably reflects the long time period covered by the collection, the large phylogenetic distance between isolates and the large number of clones collected. Most (73 %; 363 of 496) of the sites of IS-related events were unique to a single isolate (Fig. 3a). We investigated the 28 IS-related events common to at least ten isolates to identify events that had been selected through evolution. Six of these events occurred more than 100 bp away from a translation start site (Data S5). Eight of the 23 IS-related events potentially affecting gene function disrupted a gene, ten occurred close (<100 bp) to the translation start site of a single gene, and five events occurred close to the transcription start sites of two genes (Table 3). One third of the IS events in our analysis of *E. faecalis *, disrupted genes but most insertions (20 of 28) occurred in intergenic regions, as already reported in *Shigella flexneri* strain 2457T [19]. We therefore tried to determine the functions of the proteins encoded by the genes with promoters or coding sequences disrupted by an IS. A third of the IS-disrupted genes encoded proteins of unknown function (Table 3). None of the IS insertions identified affected known antibiotic resistance or virulence genes.

**Table 3.** IS-related events common to at least ten of the 168 *E. faecalis* isolates from the Raven *et al.* dataset [11]

Isolates (*n*)	IS	Position		Protein potentially affected by the IS insertion	
		**Genomic**	**In relation to the closest gene**	**Function**	**GeneID**
10	IS*Enfa4*	499 863	In	Hypothetical protein	gene540
10	IS*Efa10*	2 594 127	Upstream	HAD superfamily hydrolase	gene2620
			Downstream	Hypothetical protein	gene2621
10	IS*6770*	1 627 490	In	ABC transporter ATP-binding protein	gene1652
10	IS*Enfa4*	705 382	Downstream	cell wall surface anchor family protein	gene733
11	IS*Enfa4*	491 272	Upstream	Hypothetical protein	gene530
11	IS*1062*	991 057	In	Phosphorylase	gene1006
11	IS*Enfa4*	2 443 962	Downstream	Conjugal transfer protein	gene2462
12	IS*Chh1*	1 337 504	Upstream	Hypothetical protein	gene1348
			Downstream	hydroxymethylglutaryl-CoA synthase	gene1349
13	IS*Enfa3*	1 224 046	Downstream	ABC transporter ATP-binding protein	gene1240
14	IS*Efa10*	218 868	Upstream	Hypothetical protein	gene222
14	IS*6770*	832 847	Upstream	Potassium uptake protein	gene850
19	IS*Efa10*	2 650 256	Upstream	Lipoate-protein ligase A	gene2676
21	IS*Efa10*	2 594 120	Downstream	HAD superfamily hydrolase	gene2620
			Upstream	Hypothetical protein	gene2621
21	IS*Efa10*	2 809 601	Downstream	Hypothetical protein	gene2863
			Upstream	Valyl-tRNA synthetase	gene2864
29	IS*Apl3*	352 544	In	DadA family oxidoreductase	gene418
31	IS*Efa10*	2 404 627	Upstream	Hypothetical protein	gene2430
32	IS*Efm1*	1 317 331	In	Hypothetical protein	gene1332
34	IS*Efa10*	2 594 130	Downstream	HAD superfamily hydrolase	gene2620
			Upstream	Hypothetical protein	gene2621
37	IS*Enfa4*	1 954 670	In	Hypothetical protein	gene1987
39	IS*1485*	1 805 579	In	3-methyl-2-oxobutanoate hydroxymethyltransferase	gene1826
43	IS*Efa4*	608 941	In	DeoR family transcriptional regulator	gene644
43	IS*6770*	641 514	Upstream	Rotamase	gene672

The second and third columns give the name of the IS and the position of its insertion in the reference genome *E. faecalis V583*. The fourth column gives the position of the IS insertion in relation to the gene potentially affected, and the fifth column gives the function of the protein and the gene ID of the gene potentially affected by the IS. More detailed information are given in Data S5.

We then explored the distribution of IS-related events in the genomes of *E. faecalis.* Surprisingly, 21 % of these events (281 of 1348) were clustered together in a 150 kb region in which the IS insertion frequency (1.8 insertions/1000 bp) was four times that elsewhere in the genome (0.4 insertions/1000 bp) (Fig. 3b, green sector). We also analysed GC-content in the genome and found that this region had a lower GC-content that the rest of the genome, indicating recent horizontal gene transfer [19]. This region is a pathogenicity island (PAI) encompassing genes encoding the cytolysin toxin, the enterococcal surface protein Esp, Gls-24-like proteins, and proteins of unknown function [20–23] (Fig. 3b). Overall, 74 % of the genomes studied (125 of 168) presented at least one IS-related event in this 150 kb region, despite the uneven coverage of this region between genomes. The accelerated genetic drift of this PAI might suggest a neutral or positive biological impact of gene disruption but needs further exploration. The gene content of this PAI is known to be highly variable, but the effect of IS insertion on the evolution of this region has yet to be explored [24]. However, ISs have been implicated in the diversification of *E. faecium* [25]. Conversely, we also identified a region (between positions 2 204 066 and 2 258 320 of the reference genome *E. faecalis* V583) with a higher GC-content (Fig. 3b, red sector). This region corresponds to an integrative conjugative element (ICEEEfaV583-1) encompassing the vancomycin resistance cassette *vanB.* The detection of IS in that particular region was impossible since absent in all genomes in this dataset but one.

### Limitations and benefits of *panISa*

The choice of reference genome impacts the number of ISs detected, with the background noise increasing with the distance between the studied sequences and the reference genome. Moreover, the detection of IS in a genomic region absent from the reference genome is impossible with *panISa. PanISa* requires manual curation of the annotation after finding/validating the IS in the genomes. As the *panISa* pipeline runs with raw reads as an input, it can expedite the reanalysis of sequenced data, avoiding the step of assembly. The validation of the IS-related events is based on the reconstructed boundaries of the IS (i.e. the IR), therefore IS fragments are also detected and reported. *PanISa* is easy to install and requires few dependencies, is lightweight and can be run on a laptop, making server implementation unnecessary.

The dynamics of IS insertion within bacterial genomes remains incompletely understood, but several studies have shown that the sites of transposable element insertion are not randomly distributed between coding and non-coding regions [19, 26]. We show here that IS insertions are not randomly distributed throughout the genome. The detection of these events with *panISa* software will shed light on the dynamics of IS insertion.

### Conclusion

*PanISa* is a software pipeline for detecting IS insertions in prokaryotic genomes from short-read data. It expands the toolkit available for exploring the evolution of prokaryotic lineages. ISs are difficult to detect in short-read sequencing data, and this has probably resulted in an underestimation of the impact of these mobile elements on the evolution of their bacterial hosts. Studies of IS dynamics in bacterial genomes have also been hindered by difficulties in genome assembly.

*PanISa* can increase our understanding of the evolution of bacterial pathogens during their spread. For example, we show here that *panISa* can consolidate phylogenetic analyses of large datasets. It can also accelerate the identification of IS events with a known biological impact, such as those triggering resistance to third-generation cephalosporins in *A. baumannii. PanISa* can also be used to identify new functional impacts of ISs during the spread of pathogens. Determinations of the pattern or frequency of IS insertion over the genome will undoubtedly help us to decipher the evolution of bacterial lineages and the dynamics of IS insertions.

## Data Bibliography

1. Eppinger M, Pearson T, Koenig SSK, Pearson O, Hicks N, *et al.* Genomic epidemiology of the Haitian cholera outbreak: a single introduction followed by rapid, extensive, and continued spread characterized the onset of the epidemic. *mBio* 2014;5:e01721-14.

2. Martinez-Urtaza J, van Aerle R, Abanto M, Haendiges J, Myers RA, *et al*. Genomic variation and evolution of *Vibrio parahaemolyticus* ST36 over the course of a transcontinental epidemic expansion. *mBio* 2017;8:e01425-17.

3. Raven KE, Reuter S, Gouliouris T, Reynolds R, Russell JE, *et al*. Genome-based characterization of hospital-adapted *Enterococcus faecalis* lineages. *Nature Microbiology* 2016;1:15033.

4. Wilson MR, Brown E, Keys C, Strain E, Luo Y, *et al*. Whole genome DNA sequence analysis of *Salmonella* subspecies enterica serotype Tennessee obtained from related peanut butter foodborne outbreaks. *PLOS ONE* 2016;11:e0146929.

5. Holt K, Kenyon JJ, Hamidian M, Schultz MB, Pickard DJ, *et al*. Five decades of genome evolution in the globally distributed, extensively antibiotic-resistant *Acinetobacter baumannii* global clone 1. *Microbial Genomics*;2. Epub ahead of print 9 February 2016. DOI: 10.1099/mgen.0.000052.

## Supplementary Data

Supplementary material 1
